# Change in the Embedding Dimension as an Indicator of an Approaching Transition

**DOI:** 10.1371/journal.pone.0101014

**Published:** 2014-06-30

**Authors:** Yair Neuman, Norbert Marwan, Yohai Cohen

**Affiliations:** 1 Ben-Gurion University of the Negev, Be'er Sheva, Israel; 2 Potsdam Institut for Climate Impact Research, Potsdam, Germany; 3 Gilasio Coding, Tel-Aviv, Israel; University of Maribor, Slovenia

## Abstract

Predicting a transition point in behavioral data should take into account the complexity of the signal being influenced by contextual factors. In this paper, we propose to analyze changes in the embedding dimension as contextual information indicating a proceeding transitive point, called OPtimal Embedding tRANsition Detection (OPERAND). Three texts were processed and translated to time-series of emotional polarity. It was found that changes in the embedding dimension proceeded transition points in the data. These preliminary results encourage further research into changes in the embedding dimension as generic markers of an approaching transition point.

## Introduction

When observing a time-series, it is important to predict significant changes such as the burst of an epidemic [1], the collapse of a political regime [2], or the change in a person's mood. In recent years there have been intensive efforts in identifying early-warning signals of an approaching tipping-point [3]–[5]. While several generic signals have been identified, it was recently argued [6] that there is “no single best indicator or method for identifying an upcoming transition” and that “all methods required specific data-treatment to yield sensible signals”. Therefore, there is no single and simple generic indicator of an approaching-tipping point. This conclusion probably holds for non-catastrophic transitions [7] that are much more frequent than catastrophic transitions. Moreover, in a recent comment published in Nature, Boettiger and Hastings argue that “Truly generic signals warning of tipping points are unlikely to exist” and that researchers should study “transitions specific to real systems” [8].

The above qualifications and suggestions, may be highly relevant to the behavioral and social sciences where the signal (e.g., the mood of a person) is embedded in a complex context that may be difficult formalizing for predicting approaching transitions. In other words, the complexity of a behavioral signal is probably embedded in the context in which the signal unfolds. For instance, it was recently argued that timing of violent protests in the Middle East and North Africa can be explained by large peaks in global food prices [9]. However, the fact that violent protests were not evident everywhere in this region suggests that there are contextual factors moderating the negative influence of this increase. The contextual nature of transitions in behavioral signals (e.g., [10], [11]) invites novel approaches for predicting transitions.

In this paper, we would like to introduce a novel indicator of an approaching transition in complex behavioral data and to test it on three time-series involving mood change in textual data. The results support our hypothesis and invite further research on the issue.

## Methods and Materials

### Change in the embedding dimension as an indicator of an approaching transition

When analyzing a time-series, we usually consider it through the lenses of low-dimensionality assuming the originating system is “living” in a low-dimensional space. However, it is possible that what we observe is a projection of a system living in a higher-dimensional space [12]. This idea is highly relevant for the behavioral and social sciences where the “complexity” of an observed signal is explained by its “contextual” nature. The idea of “context”, which is the *sine qua non* of the behavioral sciences can here be interpreted as the dimensionality in which the signal unfolds. Therefore, a change in the dimensionality of a system may be indicated by a change of the embedding dimension necessary for unfolding the dynamics represented by a time-series. Such increase or decrease of the embedding dimension is actually a change in the *complexity of the context* that influences the behavior of the system. To test this hypothesis, we analyzed the time-series extracted from three different texts.

### Data and pre-processing

Three texts were selected and transformed into time series. The first text is the novel “The Jungle” (abbreviated as JUNG) written in 1906 by the American Novelist Upton Sinclair [13]. The book depicts poverty, the absence of social programs, unpleasant living and working conditions, and the hopelessness prevalent among the working class. The second text is the transcript of the romantic comedy film “When Harry Met Sally …” (1989) (abbreviated as HS) which is rated to be among the Top-10 romantic comedies of all times. The third text is a “manifesto” (abbreviated as MAN) written by a mass-shooter, an ex-policeman, by the name of Richard Dorner, for explaining his reasons for acting violently against people. The texts we have chosen represent different genres but in all of them we've expected to find significant fluctuations in the polarity of mood as they are emotionally loaded.

### Preprocessing

Each text was automatically analyzed in several phases according to common procedures used in natural language processing. These phases are presented and illustrated through a toy example.

First, we used a Part-of-Speech Tagger [14] and automatically identified words belonging to four part of speech categories: nouns, verbs, adjectives, and adverbs. Words that were not tagged as belonging to these categories, punctuation marks etc. have been removed. For example, let us analyze the following two sentences:

It was a sunny day and the friendly child travelled in the green yard. Suddenly he heard a frightening voice and noticed that a vicious looking and violent dog is barking behind the fence.

Identifying words belonging to the abovementioned speech categories we get the following output:

sunny day friendly child travelled green yard. Suddenly heard frightening voice noticed vicious looking violent dog barking fence.

Next, we use a lemmatiser (BioLemmatizer 1.1. http://biolemmatizer.sourceforge.net/). The lemmatiser automatically derives the base form (lemma) of words. For the above sentences there are four words that have been converted into a base form:

Traveled 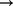 travel
Heard 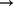 hear
Frightening 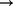 frighten
Barking 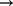 Bark

The number of unique words in each text we have analyzed were 6,009 for JUNG, 1,208 for MAN, and 915 for HS.

Next we measured the “semantic orientation” of each word. The evaluative character of a word is called its semantic orientation. Semantic orientation varies in both direction (positive or negative) and degree (mild to strong) and can serve as an indicator of the words' *general emotional polarity* (positive vs. negative).

We have used a method for inferring the semantic orientation of a word from its statistical association with a set of positive and negative paradigm words [15] and measured the semantic orientation of each word. Practically, this phase involves measuring the semantic distance of each word from the list of paradigm positive words 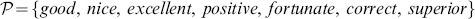 minus its distance from the list of paradigm negative words 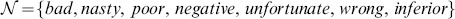.

The semantic distance between two words is calculated using the mono matrix 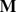 that is a 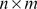 matrix of joint probabilities of 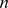 words and 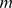 preceding/following words (details can be found in [15]). The row 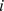 of 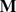 forms a vector 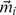 that contains probabilities that the word 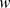 (corresponding to row 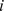) appears before or after the word belonging to a certain column. The semantic distance 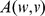 is then simply the cosine of the angle between the vectors 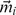 and 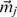 (where row 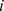 corresponds to word 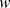 and 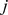 corresponds to word 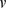): 
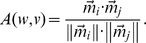



In order to calculate the semantic distance between a word 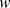 and the paradigm positive/negative words 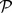 and 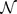, we sum up the similarity vectors 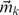 of the words belonging to those sets, i.e., 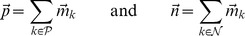
and calculate



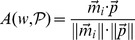
(and analogously 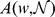). The semantic orientation 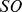 of the word 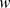 is finally the difference



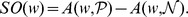



For the above toy sentences the scores 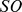 are:



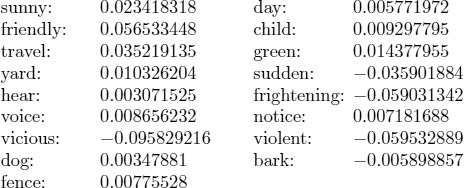



We can see that the words that got the highest positive scores in the above example were: *travel* and *sunny* while the words that got the most negative scores were *vicious* and *violent*.

To represent each text as a time series, we simply represent the words as one continuous string of scores according to the word order in the text. Using the above example the produced time series is: 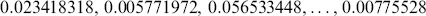. Notice that the data point is a positive number if the semantic orientation is positive and otherwise negative.

**Figure 1:**
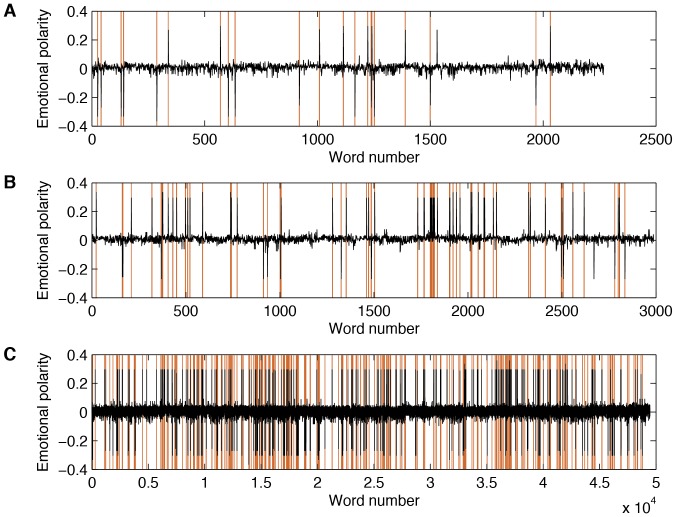
Data and transition points for (A) MAN, (B) HS, and (C) JUNG.

Applying this procedure to our texts, we produced a time-series of 49,466 data points for JUNG, 2,987 for HS and 2,269 for MAN. Fig. 1 presents the time-series of each text.

The onset of a transition point from the time series 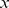 is detected as the time where the first derivative exceeds a certain threshold. Here we chose 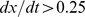. This resulted in 40 transition points for MAN, 123 for HS, 697 transitions for JUNG (Tab. 1).

**Table 1:** Data, data length, and found number of transitions.

Dataset	Data length	Number transitions
MAN	2,269	40
HS	2,987	123
JUNG	49,466	697

### Estimating dimensionality

For the estimation of the dimensionality change of the system we are using embedding dimension and check it with another measure reflecting system's dimensionality, the recurrence network transitivity.

The first measure attempts to estimate the optimal embedding dimension 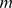 from a time series by using the false nearest neighbours approach [16]. A phase space embedding assumes that the state 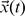 of a 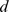-dimensional dynamical system, which is represented by its 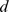 state variables 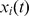 (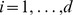), can be reconstructed from only one observed variable, e.g., 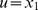, by using time-delay embedding [17], 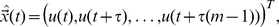
where 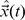 is the reconstructed phase space trajectory of the system, topologically equivalent to the original 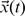, 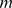 is the embedding dimension, and 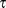 the time-delay. The idea of the false nearest neighbours approach is that a phase space vector 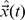 can have false neighbours when the dimension of the phase space is not sufficient. We count the amount of false neighbours in the phase space for increasing embedding dimension 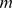. We assume, that such embedding dimension is optimal when the amount of false neighbours vanishes. Changes in the embedding dimension over time can be used to study dynamical transitions. We propose this method as an *OPtimal Embedding tRANsition Detection* (OPERAND) approach.

The second approach is based on a recently introduced novel dimensionality measure which is based on geometrical and recurrence properties in the phase space. A recurrence plot 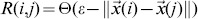 of the phase space vectors [18] is considered to be the adjacency matrix of a complex network [19], [20]. In the following we consider the discretized time 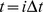, where 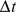 is the sampling time and 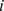 is the time index in the time-series. We calculate then the *transitivity coefficient* 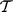


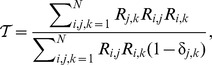
of this recurrence network. A dimensionality measure can then be defined by [21]



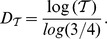



This allows the calculation of the dimension without explicit consideration of scaling behaviours.

We calculate the optimal dimension and the transitivity dimension from subsequences of the data of length 100 data points. We distinguish two sets of such subsequences: (A) the first set contains the subsequence just before the onset of the transition point. (B) the second set contains the subsequences of the data where the period before and after the onset is excluded (we consider it as the reference data set). The length of the excluded part is twice the length of the subsequences, where the onset time point is in the middle of the removed part. Calculation in the reference part is applied using sliding windows with moving step of 20 data points, allowing for more calculations.

Finally we compare the distributions of the two dimensionality measures for the two sets (A) and (B) of the subsequences. We use the Wilcoxon rank-sum test to statistically test the difference of the median of the detected dimensions between the two sets (A) and (B).

## Results

**Figure 2:**
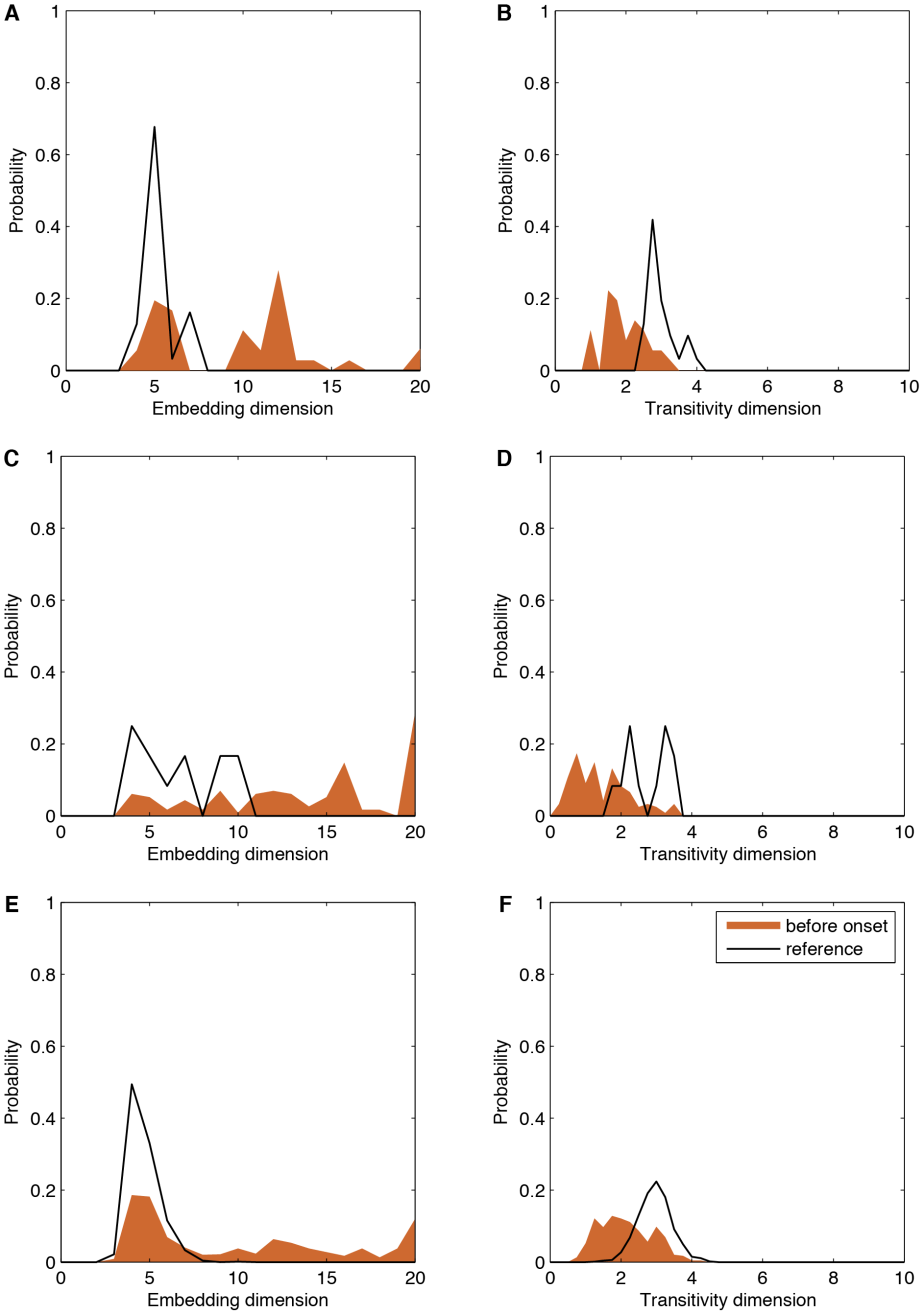
(A, C, E) Embedding dimensions and (B, D, F) and transitivity dimensions for (A, B) MAN, (C, D) HS, and (E, F) JUNG data series.

Based on the OPERAND approach we find significantly higher embedding dimensions 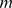 for the epochs before the onset of the transition point than for the remaining period (Tab. 2, Figs. 2).

**Table 2:** Median values of the optimal embedding dimension 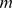 and transitivity dimension 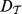 for the considered data sets before transition point onset and for the reference period.

Measure		MAN	HS	JUNG
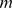	before onset	10	15	8
	reference	5	6.5	4
	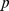 -value	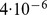	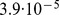	0.000
				
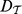	before onset	1.9	1.3	2.1
	reference	2.8	2.8	3
	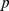 -value	0.000	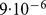	0.000

**Figure 3:**
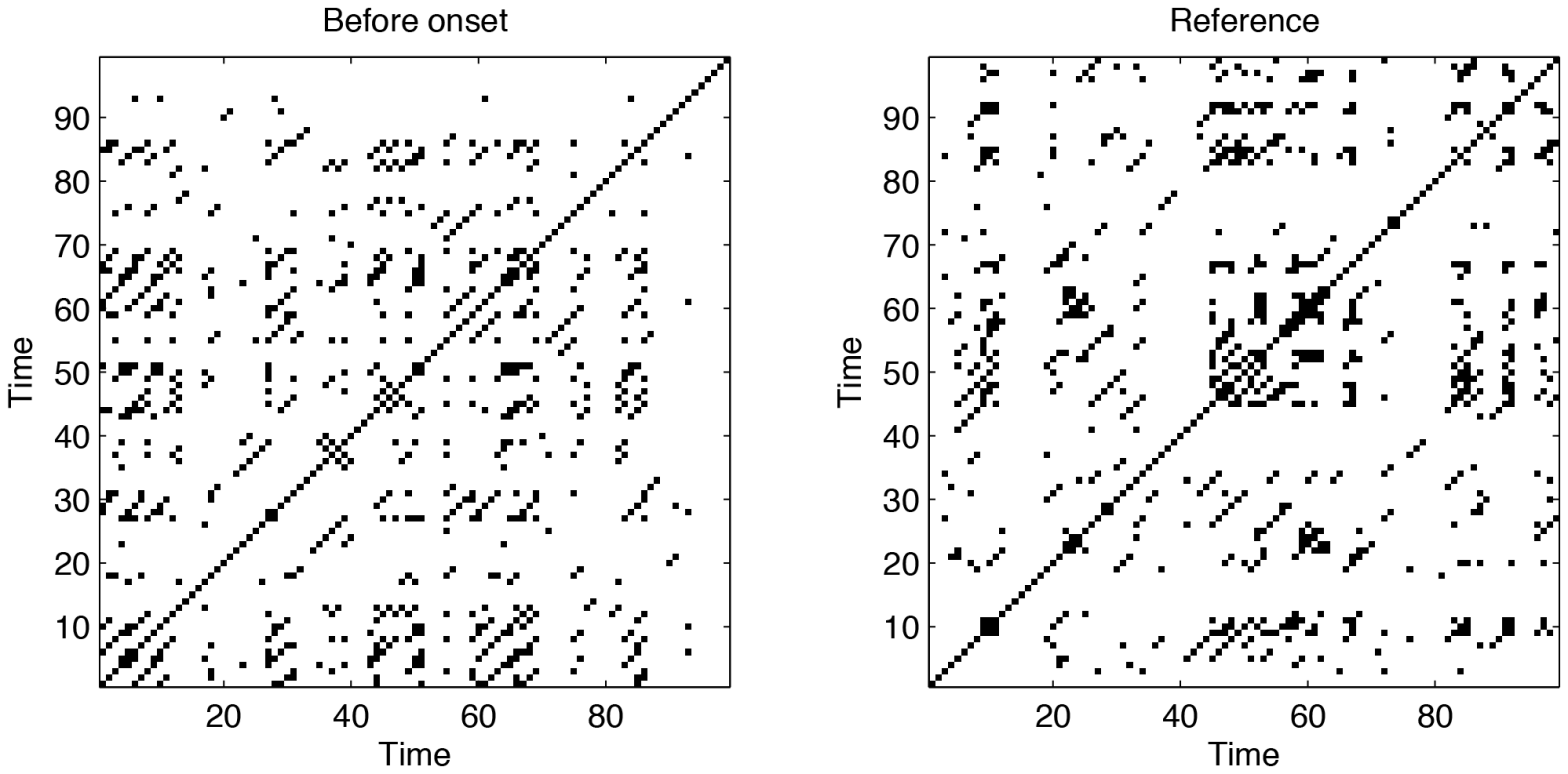
Exemplary recurrence plot before transition point onset (left) and the reference period (right). Embedding dimension 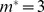, embedding delay 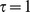, recurrence threshold 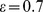, window size 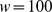.

The recurrence plot is calculated using an embedding dimension 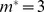 and a recurrence threshold of 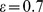. Fig. 3 illustrates exemplary recurrence plots before transition point onset and the reference period. After removing the main diagonal, the transitivity dimension 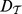 is calculated. We find that 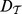 is significantly lower before the onset, than for the reference period.

The difference between the medians of the dimension values is highly significant: for all data sets the 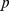 -values are below 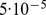.

Before transition onset, the embedding dimension increased, whereas the transitivity dimension counterintuitively decreased. This points to a general problem, often neglected when investigating transitions in dynamical systems using phase space reconstruction. For the transitivity dimension we have used fixed embedding parameters. Therefore, just before the onset, the dynamics is embedded in a too small phase space. Therefore, the transitivity dimension reveals a smaller value than in the correctly embedded reference period. We have tested this effect using a dynamical embedding, where we have applied an optimal embedding dimension (as it comes from OPERAND) for each sliding window. Then the transitivity dimension shows the same behavior as the embedding dimension test.

## Conclusions

In this paper, we introduce a new method for identifying an approaching transition in behavioral data. The idea is that the complexity of behavioral signals usually resides in what social scientists describe as “context”, or what [22] in his classical work describes as the totality of signals that directed the behavior of the organism. A transition in the behavior of the signal is expected if the context in which this signal is embedded undergoes changes in itself. Using changes in the embedding dimension as an indication of an approaching transition is therefore a shift from focusing on the dynamics of the signal to the dynamics of the meta-system in which it is subordinated. This idea is here tested for the first time and currently under further developments. We also see a wide applicability of the suggested optimal embedding transition detection (OPERAND) approach. Changes in embedding as well as transitivity dimension might also be able to detect important transition points, e.g, in the climate system or in financial markets [5].
